# Supplementary material for: Pseudo-topotactic conversion of carbon nanotubes to T-carbon nanowires under picosecond laser irradiation in methanol
Source: Nat Commun. 2017 Sep 25;8:683. doi: 10.1038/s41467-017-00817-9 (PMC5612968; doi:10.1038/s41467-017-00817-9)
Supplement: Supplementary file 1 — Supplementary Information [file 41467_2017_817_MOESM1_ESM.pdf]

Description of Supplementary Files

File Name: Supplementary Information
Description: Supplementary Figures, Supplementary Tables, Supplementary Methods and Supplementary References

File Name: Peer Review File

Supplementary Methods

MWCNT cutting. The MWCNTs were shortened by a sonication method according to the reported literature.[1] Briefly, 50 mg MWCNTs were suspended in toluene (99.5%, Xilong Scientific) via a water bath sonication. The solution was then centrifuged at 6000 rpm (9483×g) for 10min at room temperature to remove the toluene with carbon impurities. The nanotubes were then dispersed in 40ml of heptane (97%, Sinopharm) to which 20ml of 0.5 M HCl solution was added by a ultrasonication method using a cell disruptor S-450D (Branson, tip: 1/8'') for 2min at an interval of 5s at the power of 50W. The dark aggregate was then washed twice by 40 mL milliQ-water and re-suspended in 40 mL heptane. This prewashing step helps to remove most of the unknown impurities and enables the aggregate to disperse more easily. 10ml of KMnO4 (99.5%, Sinopharm) water solution (10 mg/ml) was then added to the prewashed MWCNTs suspension in 40 mL heptane. The mixture was further sonicated for 10min with the same tip and parameters. The aqueous phase was then decanted. 40 mL of 0.5 M H_2SO4 was then added to the heptane phase and sonicated for 2min to remove the MnO_2. Extra H_2SO_4 was used to remove the metal impurities and then washed by milli-Q water. The whole shortening sequence was repeated twice. The shortened MWCNTs suspension was then filtered by a 0.45 μm membrane film and thoroughly rinsed by acetone. The MWCNTs were then dried in an oven overnight.

Extinction rules for T-carbon. The structure factor is defined below, where x_j, y_j, and z_j are the coordinates in terms of lattice vectors of each of the atoms in the basis:

$$F_{hkl} = \sum_{j=1}^{N} f_j e^{-i2\pi\left(x_j h + y_j k + z_j l\right)} \tag{S1}$$

where f is the atomic form factor for T-carbon; the cubic cell has a 32-atom basis for which (x_i, y_i, z_i) are:

(1/2+a, 1/2-a, 1-a); (1/2-a, a, 1/2-a); (1-a, 1-a, a); (a, 1/2+a, 1/2+a);

(a, a, a); (1-a, 1/2+a, 1/2-a); (1/2-a, 1/2-a, a); (a, 1-a, 1-a)

(1/2+a, a, 1/2+a); (1/2-a, 1-a, 1/2+a); (a, 1/2-a, 1/2-a); (1-a, a, 1-a)

(1/2+a, 1/2+a, a); (1/4-a, 3/4+a, 3/4+a); (3/4+a, 3/4-a, 1/4+a) (3/4-a, 1/4-a, 3/4-a)

(1/4+a, 1/4+a, 1/4-a); (1/4+a, 1/4-a, 1/4+a); (1/4-a, 3/4-a, 3/4-a); (1/4-a, 1/4-a, 1/4-a)

(3/4-a, 1/4+a, 3/4+a); (3/4+a, 3/4+a, 1/4-a); (3/4-a, 3/4-a, 1/4-a); (1/4-a, 1/4+a, 1/4+a)

(3/4+a, 1/4-a, 3/4+a); (1/4+a, 3/4+a, 3/4-a); (3/4-a, 3/4+a, 1/4+a); (1/4+a, 3/4-a, 3/4+a)

(3/4+a, 1/4+a, 3/4-a); (1/2-a, 1/2+a, 1-a); (1/2+a, 1-a, 1/2-a); (1-a, 1/2-a, 1/2+a)

where a = 0.0706 is a constant.

The structure factor of T-carbon can be written as **Equation S2:**

$$
\begin{aligned}
F_{hkl} = f[& e^{-i2\pi\left((\frac{1}{2}+a)h+(\frac{1}{2}-a)k+(1-a)l\right)} + e^{-i2\pi\left((\frac{1}{2}-a)h+ak+(\frac{1}{2}-a)l\right)} + e^{-i2\pi\left((1-a)h+(1-a)k+al\right)} + e^{-i2\pi\left(ah+(\frac{1}{2}+a)k+(\frac{1}{2}+a)l\right)} \\
& + e^{-i2\pi\left(ah+ak+al\right)} + e^{-i2\pi\left((1-a)h+(\frac{1}{2}+a)k+(\frac{1}{2}-a)l\right)} + e^{-i2\pi\left((\frac{1}{2}-a)h+(\frac{1}{2}-a)k+al\right)} + e^{-i2\pi\left(ah+(1-a)k+(1-a)l\right)} \\
& + e^{-i2\pi\left((\frac{1}{2}+a)h+ak+(\frac{1}{2}+a)l\right)} + e^{-i2\pi\left((\frac{1}{2}-a)h+(1-a)k+(\frac{1}{2}+a)l\right)} + e^{-i2\pi\left(ah+(\frac{1}{2}-a)k+(\frac{1}{2}-a)l\right)} + e^{-i2\pi\left((1-a)h+ak+(1-a)l\right)} \\
& + e^{-i2\pi\left((\frac{1}{2}+a)h+(\frac{1}{2}+a)k+al\right)} + e^{-i2\pi\left((\frac{1}{4}-a)h+(\frac{3}{4}+a)k+(\frac{3}{4}+a)l\right)} + e^{-i2\pi\left((\frac{3}{4}+a)h+(\frac{3}{4}-a)k+(\frac{1}{4}+a)l\right)} + e^{-i2\pi\left((\frac{3}{4}-a)h+(\frac{1}{4}-a)k+(\frac{3}{4}-a)l\right)} \\
& + e^{-i2\pi\left((\frac{1}{4}+a)h+(\frac{1}{4}+a)k+(\frac{1}{4}-a)l\right)} + e^{-i2\pi\left((\frac{1}{4}+a)h+(\frac{1}{4}-a)k+(\frac{1}{4}+a)l\right)} + e^{-i2\pi\left((\frac{1}{4}-a)h+(\frac{3}{4}-a)k+(\frac{3}{4}-a)l\right)} + e^{-i2\pi\left((\frac{1}{4}-a)h+(\frac{1}{4}-a)k+(\frac{1}{4}-a)l\right)} \\
& + e^{-i2\pi\left((\frac{3}{4}-a)h+(\frac{1}{4}+a)k+(\frac{3}{4}+a)l\right)} + e^{-i2\pi\left((\frac{3}{4}+a)h+(\frac{3}{4}+a)k+(\frac{1}{4}-a)l\right)} + e^{-i2\pi\left((\frac{3}{4}-a)h+(\frac{3}{4}-a)k+(\frac{1}{4}-a)l\right)} + e^{-i2\pi\left((\frac{1}{4}-a)h+(\frac{1}{4}+a)k+(\frac{1}{4}+a)l\right)} \\
& + e^{-i2\pi\left((\frac{3}{4}+a)h+(\frac{1}{4}-a)k+(\frac{3}{4}+a)l\right)} + e^{-i2\pi\left((\frac{1}{4}+a)h+(\frac{3}{4}+a)k+(\frac{3}{4}-a)l\right)} + e^{-i2\pi\left((\frac{3}{4}-a)h+(\frac{3}{4}+a)k+(\frac{1}{4}+a)l\right)} + e^{-i2\pi\left((\frac{1}{4}+a)h+(\frac{3}{4}-a)k+(\frac{3}{4}+a)l\right)} \\
& + e^{-i2\pi\left((\frac{3}{4}+a)h+(\frac{1}{4}+a)k+(\frac{3}{4}-a)l\right)} + e^{-i2\pi\left((\frac{1}{2}-a)h+(\frac{1}{2}+a)k+(1-a)l\right)} + e^{-i2\pi\left((\frac{1}{2}+a)h+(1-a)k+(\frac{1}{2}-a)l\right)} + e^{-i2\pi\left((1-a)h+(\frac{1}{2}-a)k+(\frac{1}{2}+a)l\right)}]
\end{aligned}
$$

(S2)

Since $e^{-i\pi(2n)}$=1 when n is integer, **Equation S1** can be simplified to **Equation S3:**

$$F_{hkl} = f[1+(-1)^{h+k}+(-1)^{h+l}+(-1)^{k+l}][1+(-i)^{h+k+l}]\chi(a,h,k,l) \qquad \text{(S3)}$$

If *h, k, l* are of mixed parity (odd and even values combined) the first term is zero, so $|F_{hkl}|^2 = 0$

If *h, k, l* are either all even or all odd, then the first term $[1+(-1)^{h+k}+(-1)^{h+l}+(-1)^{k+l}] = 4$

If *h,k,l* are all odd, $F_{hkl} = 4f(1 \pm i)$, then $|F_{hkl}|^2 = 32f^2$

If *h,k,l* are all even whose sum is a multiple of 4 ($h + k + l = 4$ n), then $F_{hkl} = 4f \times 2$. Thus $|F_{hkl}|^2 = 64f^2$

If *h,k,l* are all even whose sum is not a multiple of 4 ($h + k + l \neq 4$ n), then second term is zero. Thus $|F_{hkl}|^2 = 0$

Entropy calculation. The thermal properties of solids at constant volume can be calculated from their phonon density of states as a function of frequencies within the quasi-harmonic approximation.[2]

The phonon contribution to the Helmholtz free energy is given by:

$$F_{phonon} = \frac{1}{2}\sum_{q,v} \hbar\omega_{q,v} + k_B T \sum_{q,v} \ln[1 - \exp\left(-\frac{\hbar\omega_{q,v}}{k_B T}\right)] \tag{S4}$$

Where q and v are the wave vector and band index, respectively, $\omega_{q,v}$is the phonon frequency at q and T is the temperature. k_B and $\hbar$ are the Boltzmann constant and the reduced Planck constant, respectively. The heat capacity C_V and the entropy S at constant volume are given by:

$$C_V = \sum_{q,v} k_B \left(\frac{\hbar\omega_{q,v}}{k_B T}\right)^2 \frac{\exp(\hbar\omega_{q,v}/k_B T)}{[\exp(\hbar\omega_{q,v}/k_B T)-1]^2} \tag{S5}$$

$$S = -k_B \sum_{q,v} \ln\left[1 - \exp\left(\hbar\omega_{q,v}/k_B T\right)\right] - \quad \frac{1}{T}\sum_{q,v} \frac{\hbar\omega_{q,v}}{\exp(\hbar\omega_{q,v}/k_B T)-1} \tag{S6}$$

In practical thermodynamic problems related to solids, the thermal properties need to be known at constant pressure. They can be calculated from the previous quantities through thermodynamic relationship. The Gibbs free energy G may be written as:

$$G(T,P) = \min_V\left[U(V) + F_{phonon}(T;V) + pV\right] \tag{S7}$$

where V and p are the volume and pressure, respectively, and $U(V)$ is the total energy of electronic structure at constant volume. The right-hand side of **Equation 4** means that, for each couple of T and p variables, the function inside the square brackets is minimized with respect to the volume. Then the heat capacity at constant pressure is derived from $G(T, p)$ by:

$$C_p(T,p) = -T\frac{\partial^2 G(T,P)}{\partial T^2} = T\frac{\partial V(T,P)}{\partial T}\frac{\partial S(T,V)}{\partial V}\Big|_{V=V(T,P)} + C_V[T, V(T,P)] \tag{S8}$$

where $V(T,P)$ is the equilibrium volume at T and p.

The computed specific heat (C_p) and entropy (S) of the graphite, diamond, CNT(6,6) and T-carbon are listed in **Supplementary Table 3**.

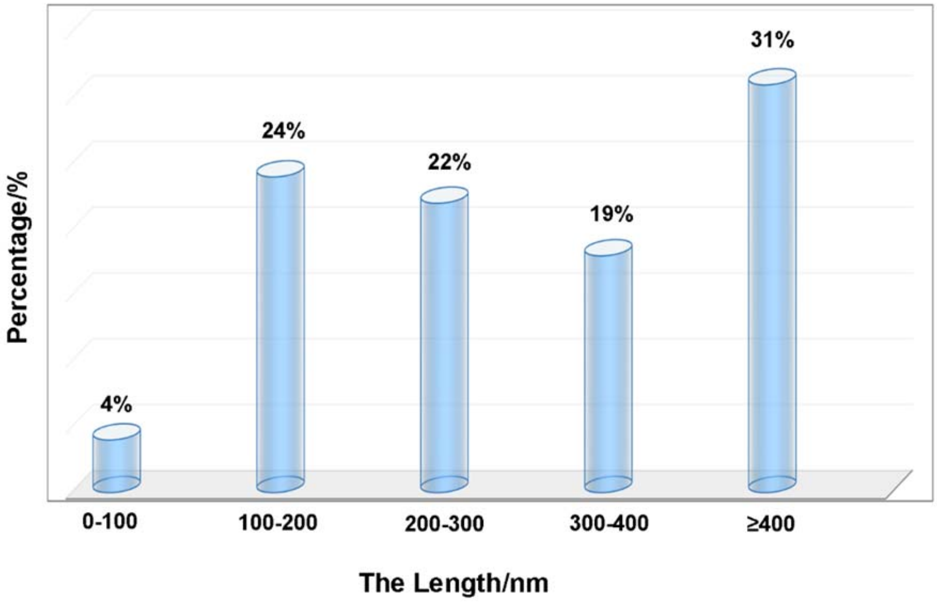

Supplementary Figure 1. Length distribution of the shortened MWCNTs.

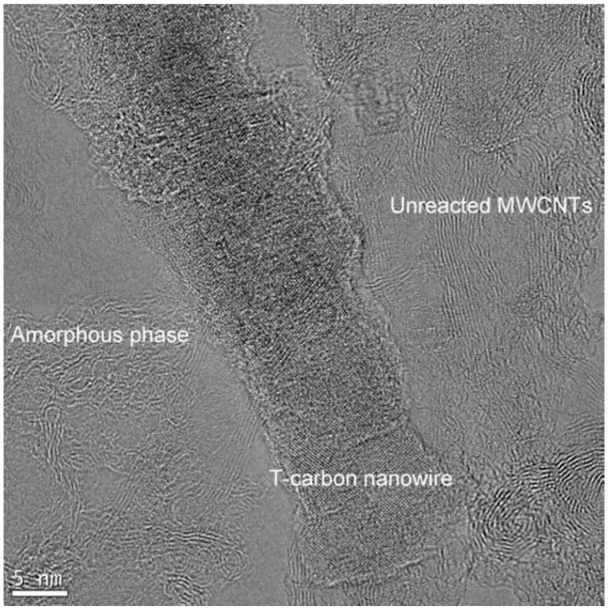

Supplementary Figure 2. HRTEM image of the products after laser irradiation.

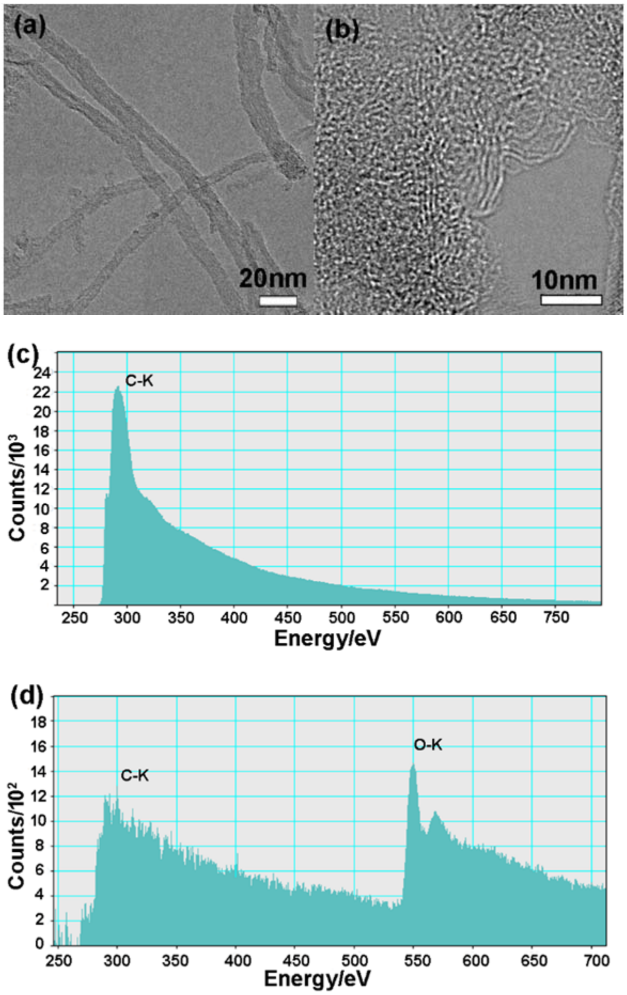

Supplementary Figure 3. HRTEM images of different products and their EELS spectra. HRTEM images of (**a**) unreacted MWCNTs and (**b**) amorphous products. EELS spectra of the (**c**) unreacted MWCNTs and (**d**) amorphous products from methanol by laser irradiation after subtraction of carbon film background.

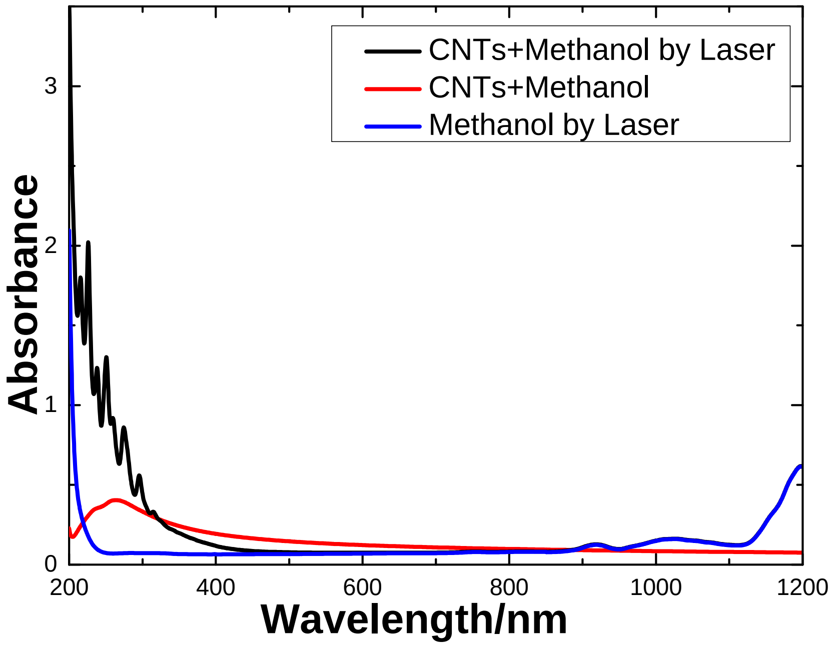

Supplementary Figure 4. UV-Vis absorption spectra of T-carbon nanowire products (black) compared with pure methanol solution after laser irradiation under the same conditions (blue) and the pristine CNTs suspension in methanol before laser irradiation (red).

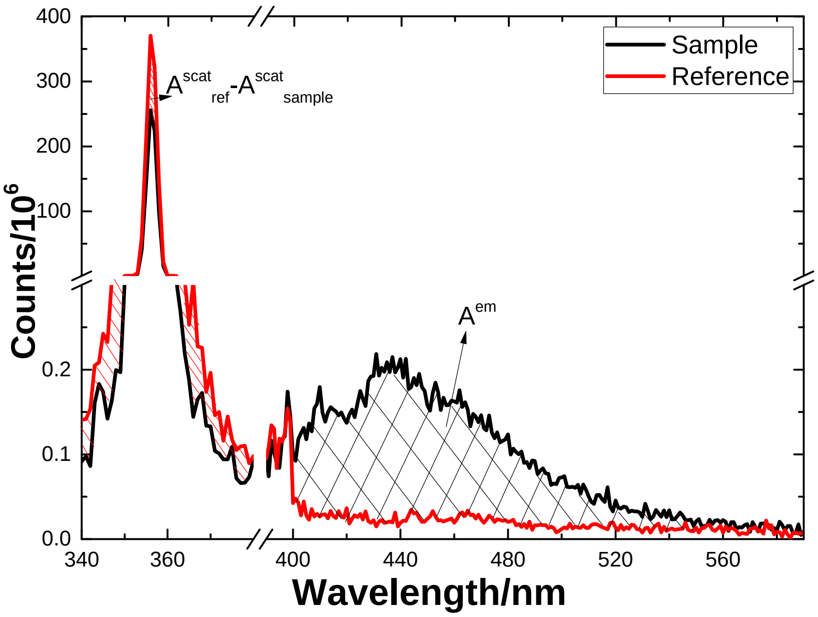

Supplementary Figure 5. Absolute photoluminescence quantum yield measurement of the T-carbon nanowires based on the crude products using an integrating sphere. The quantum yield is calculated as the ratio of the number of photons emitted (N^{em}) by the number of photons absorbed (N^{abs}). Number of emitted photons is given by the area under the spectrally corrected emission (A^{em}). Number of absorbed photons is given by the difference of areas under the Rayleigh scattering peaks of a reference sample and the sample under study ($N^{abs}=A^{scat}_{ref}-A^{scat}_{sample}$). $Q= N^{em}/ N^{abs}= A^{em}/(A^{scat}_{ref}-A^{scat}_{sample})=5.4\pm0.2\%$.[3,4]

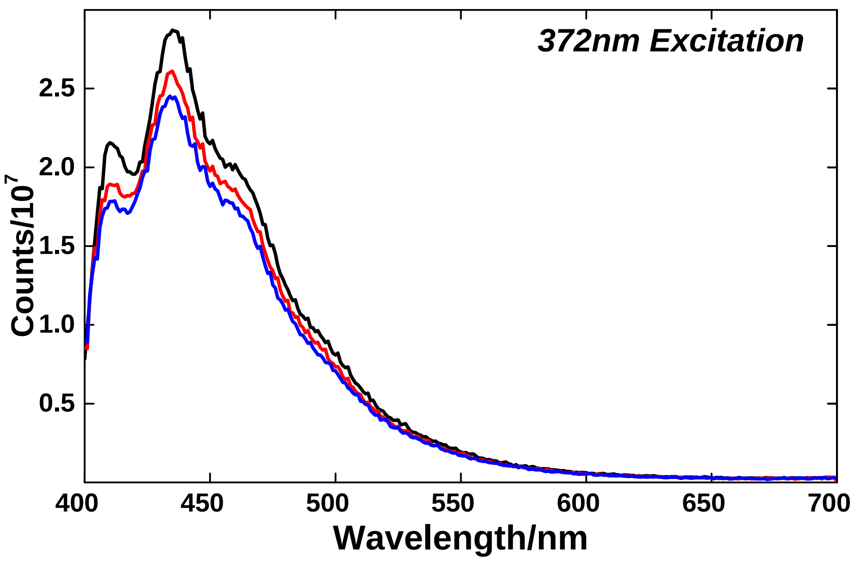

Supplementary Figure 6. Photoluminescence (PL) spectra of the same T-carbon NW suspension in methanol at different temperatures (black - 0ºC, red - 27 ºC, blue - 60 ºC).

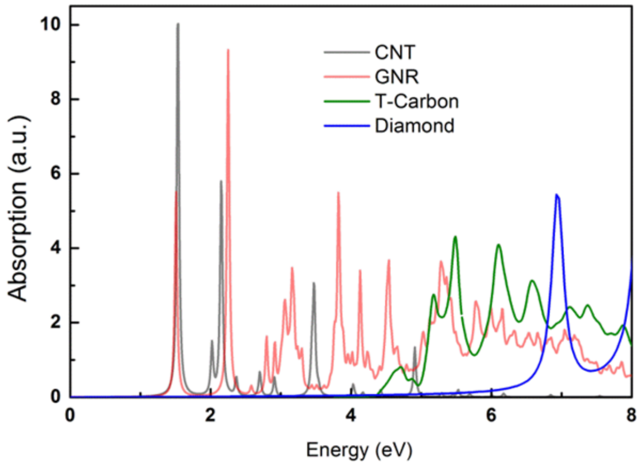

Supplementary Figure 7. Calculated absorption spectra of T-carbon, graphene nanoribbon (sp^2 carbon in GNR with a width of W14), single-walled carbon nanotube (CNT) (8,0) (sp^2-carbon in CNT), and diamond (sp^3-carbon in diamond).

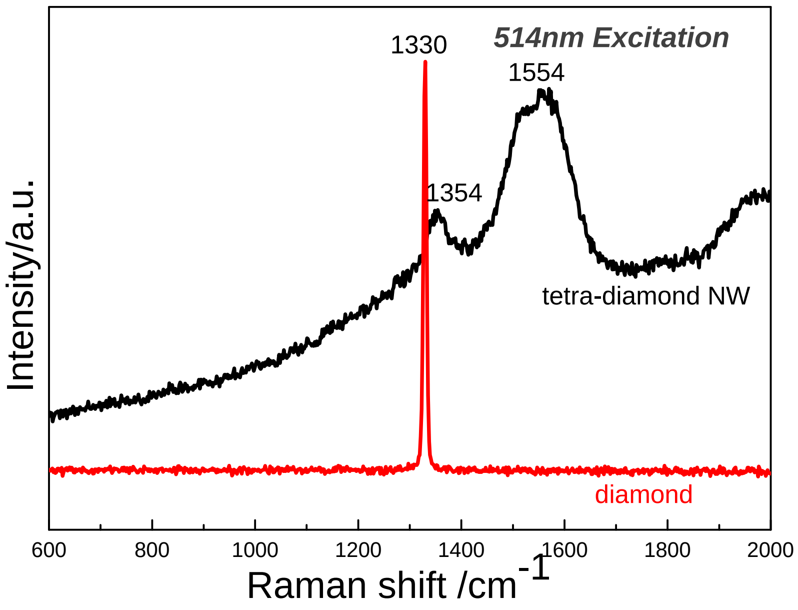

Supplementary Figure 8. Raman spectrum of the T-carbon nanowire (NW) products (black) after laser irradiation of MWCNT suspension in methanol compared with that of diamond (red).

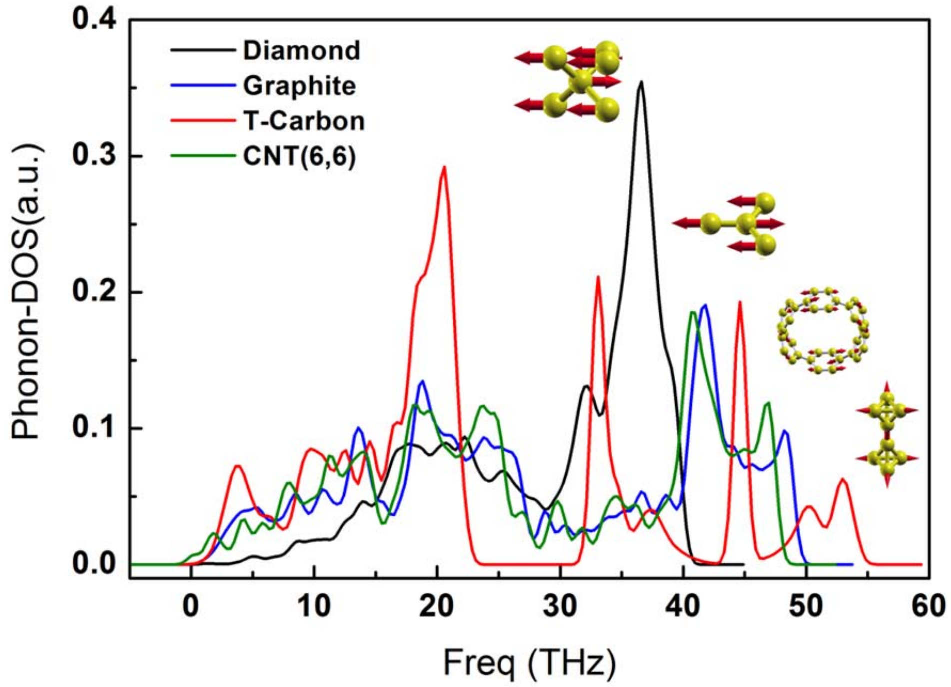

Supplementary Figure 9. Phonon density of states (Phonon-DOS) of diamond, graphite, T-carbon and CNT(6,6) respectively. The eigenvectors of high frequency longitudinal optical modes are plotted next to each structure.

Supplementary Table 1. Interplanar distances of T-carbon with a lattice constant of 7.80 Å.

{hkl}	{111}	{022}	{113}	{004}	{115}	{135}
D(Å)	4.50	2.76	2.35	1.95	1.50	1.32

Supplementary Table 2. Comparison of interplanar angles between experimental and calculated values.

hkl_1	hkl_2	$\alpha_{ex.}(°)^a$	$\alpha_{cal.}(°)^b$
0-22 (Figure 1c)	004	45±2	45.00
	022	90±2	90.00
0-22 (Figure 1d)	$\bar{2}02$	60±2	60.00
	$\bar{2}20$	120±2	120.00
0-22 (Figure 2a)	$\bar{1}\ \bar{1}3$	32±2	31.48
	$1\ \bar{3}1$	32±2	31.48
	$\bar{1}11$	90±2	90.00
004 (Figure 2b)	$1\ \bar{3}1$	72±2	72.45
	$1\ \bar{3}\ \bar{1}$	107±2	107.55
	$1\ \bar{5}\ \bar{1}$	102±2	101.10
	$1\ \bar{5}1$	80±2	78.90
	$1\ \bar{5}3$	59±2	59.53
1-31 (Figure 2b)	$1\ \bar{5}\ \bar{1}$	31±2	29.50
	$1\ \bar{5}1$	8.5±2	9.45
	$1\ \bar{5}3$	13±2	14.46

$^a\alpha_{ex.}$ refers to the experimental interplanar angles between hkl_1 and hkl_2

$^b\alpha_{cal.}$ refers to the calculated interplanar angles between hkl_1 and hkl_2

Supplementary Table 3. Specific heat (C_p) and entropy (S) of graphite, diamond, CNT(6,6) and T-carbon.

	C_p (0Pa, 300K)(J/mol/K)	S (300K) J/mol/K
Graphite	(8.25)[5] 8.53	(5.31)[6,7] (5.68)[8] 5.09
Diamond	(6.06)[9] 6.71	(2.45)[9] 2.67
MWCNT	8.04[5]	(6.43)[5] 5.85
T-carbon	10.98	7.56

Supplementary References

1. Kamalasanan, K., *et al.* "Zero-Dimensional" Single-Walled Carbon Nanotubes. *Angew. Chem. Int. Ed.* **52**, 11308-11312 (2013).

2. Togo, A. & Tanaka, I. First principles phonon calculations in materials science. *Scripta Mater.* **108,** 1-5 (2015).

3. Wuerth, C., Grabolle, M., Pauli, J., Spieles, M. & Resch-Genger, U. Comparison of Methods and Achievable Uncertainties for the Relative and Absolute Measurement of Photoluminescence Quantum Yields. *Anal. Chem.* **83,** 3431-3439 (2011).

4. Suzuki, K., Quantaurus-QY: Absolute photoluminescence quantum yield spectrometer. *Nat. Photon.* **5,** (2011).

5. Muratov, V. B., *et al.* Thermodynamic properties of multiwalled carbon nanotubes. *Journal of Superhard Mater.* **34,** 173-178 (2012).

6. Nernst, W. Der Energieinhalt fester Stoffe. *Annalen Der Physik* **36,** 395-439 (1911).

7. Koref, F. Messungen Der Spezifischen Wärme Bei Tiefen Temperaturen Mit Dem Kupferkalorimeter. *Annalen der Physik* **341,** 49-73 (1911).

8. Jacobs, C. J. & Parks, G. S. Thermal Data on Organic Compounds. Xiv. Some Heat Capacity, Entropy and Free Energy Data for Cyclic Substances. *J. Am. Chem. Soc.* **56**, 1513-1517 (1934).

9. Pitzer, K. S. The heat capacity of diamond from 70 to 300 degrees K. *J. Chem. Phys.* **6,** 68-70 (1938).
